# Risk factors of nociplastic pain in patients with autoimmune arthritis: web-based cross-sectional survey of patients

**DOI:** 10.1007/s00296-025-05948-7

**Published:** 2025-08-22

**Authors:** Grzegorz Mirocha, Joanna Makowska, Olga Brzezińska

**Affiliations:** Department of Rheumatology, Medical University of Lodz, Żeromskiego 113, Lodz, 90-549 Poland

**Keywords:** Surveys and questionnaires, Nociplastic pain, Central sensitization, Rheumatoid arthritis, Chronic pain, Non-inflammatory pain, Psoriatic arthritis, Spondyloarthropathy

## Abstract

This study aimed to identify risk factors of nociplastic pain in patients with autoimmune arthritis. Patients suffering from chronic pain and autoimmune arthritis were invited to participate in a study via moderated support groups on social media. Each invitation included a brief description of nociplastic pain. The study cohort comprised 185 patients, primarily those with rheumatoid arthritis (64%) and spondyloarthropathies (27%). 82% of patients scored positively (≥ 40 points) on the Central Sensitization Inventory (CSI), while 57% showed a neuropathic component as indicated by the PainDetect Questionnaire (PDQ). Of the participants with a positive CSI score, 86% achieved at least a borderline grade on the PDQ. More frequent use of painkillers (> once a day) correlated with higher CSI scores (mean difference (MD) = 7.1, *p* = 0.039, and MD = 8, *p* = 0.005 compared to daily and less frequent use, respectively). In a multivariable linear regression model, factors such as depression (β = 8.7, *p* < 0.001), nocturnal pain (β = 4.5, *p* = 0.013), need for painkillers (β = 5.3, *p* = 0.032), disease duration > 2 years (β = 3.3, *p* = 0.036), Chronic Pain Grade Scale (β = 2.8, from I to IV grade, *p* < 0.001), and worse mood (β = -0.9, on a 0–10 scale, *p* = 0.031) were independently associated with higher CSI scores. Nocturnal pain and pain-related disability may serve as clinical risk factors of nociplastic pain. The PDQ may lose its reliability in detecting neuropathic pain in patients with a high nociplastic component.

**Supplementary Information:**

The online version contains supplementary material available at 10.1007/s00296-025-05948-7.

## Background

Pain is a hallmark symptom of autoimmune arthritis, significantly impacting patients’ quality of life and daily functioning. It can arise from three distinct mechanisms: inflammation and tissue damage, classified as nociceptive pain, or direct damage to nerve endings, classified as neuropathic pain [1]. The third mechanism presented by the International Association for the Study of Pain (IASP) in 2016 included nociplastic pain. Nociplastic pain was defined as a pain that arises from altered nociception, which is not fully explained by nociceptive or neuropathic mechanisms. It was distinguished to describe mechanisms in primary pain syndromes such as fibromyalgia [2]. It is characterized by widespread pain, fatigue, sleep disturbances, cognitive dysfunction, depression, and anxiety. Individuals with nociplastic pain often exhibit diffuse tenderness, which indicates hyperalgesia and/or allodynia. They are often more sensitive than others to non-painful sensory stimuli such as lights, odors, and noises [3]. Central sensitization plays a leading role in developing the nociplastic pain, where changes in the central nervous system lead to amplifying neural signaling that elicits pain hypersensitivity [4, 5]. It may affect approximately 40% of patients with rheumatic diseases, influencing their reported outcomes and reducing the likelihood of achieving treatment goals [6]. This may lead to additional health and economic costs associated with treatment change, unnecessary drug intake, and more often medical consultations [7]. In 2021, the International Association for the Study of Pain (IASP) Terminology Task Force published criteria for the classification and grading of nociplastic pain manifesting within the musculoskeletal system:

1. more than 3 months duration of pain,
2. regional, multifocal, or widespread rather than discrete distribution of pain.
3. Clinical signs of hypersensitivity (e.g., mechanical or thermal allodynia or aftersensations) must be present within the region of pain.
4. The pain cannot entirely be explained by nociceptive or neuropathic mechanisms [8].

The three components of pain often coexist, leading to diagnostic and therapeutic challenges. The neuropathic component can be assessed using easily applicable and validated questionnaires, such as the PainDetect Questionnaire (PDQ) [9]. However, some authors have reported that the PDQ may overestimate the neuropathic component in primary pain syndromes [10, 11]. Our study aimed to assess lifestyle factors, treatment courses, and clinical symptoms of patients with nociplastic pain to investigate potential contributions to its development, thereby facilitating clinical screening and diagnostic approaches. Additionally, we aim to evaluate the potential utility of the PDQ in this subset of patients.

## Materials and methods

### Data collection

Invitations to participate in the study were posted from October to December 2024 to Polish social media groups gathering patients with rheumatic diseases. The invitations targeted individuals experiencing chronic pain and included a brief description of central sensitization and nociplastic pain. Each invitation was refreshed every few days until a significant response decrease was observed, or the moderator’s permission expired. The distribution of questionnaire responses over the collection period is shown in Supplementary Fig. 1. The completion of the questionnaire was limited to a single email address to prevent unintended reuse and duplicate entries. A duplicate search was also conducted during the final analysis phase. Participation in the survey was voluntary and anonymous, with no identifiable data collected from respondents. Data collection was performed using Microsoft Forms.

### Questionnaire construction and interpretation

Each questionnaire consists of five parts:

1. *Basic Personal Information*: This section gathers demographic data from participants, primary rheumatological diagnosis, disease course, treatment, and comorbidities. Each question regarding diagnosis or comorbidities included the phrase “Diagnosed by a physician” to minimize the risk of participants reporting self-diagnosed conditions, which could bias the analysis. Comorbidities were considered in the statistical analysis if the number of respondents in the subgroup was at least 20. Otherwise, it was included only to calculate the overall sum of comorbidities for each patient. The diagnostic delay was defined as the period between the first symptoms and the final diagnosis. The mood during participation was evaluated as a potential bias factor. It was measured on a visual scale ranging from 0 to 10, where 0 indicates the worst mood and 10 indicates the best.
2. *Lifestyle*: This section collects participants’ lifestyle factors, emphasizing employment, alcohol consumption (1 unit = 10 g of ethanol), cigarette smoking, and physical activity. The physical activity (PA) measure was constructed based on the recommendations of the World Health Organization (WHO) [12]. Participants were classified into four categories: no additional PA, PA below the recommended threshold, PA within 1–2 times the recommended threshold, and PA above 2 times the threshold. The quality and quantity of social interactions and support from the immediate environment were assessed on a visual scale ranging from 0 to 10, where 0 corresponds to “Very poor, I feel no support” and 10 corresponds to “Very good, I feel constant support.”.
3. *Chronic Pain Grade Scale (CPGS)*: The CPGS assesses two dimensions of overall chronic pain severity: pain intensity and pain-related disability. Those combined enable classification of chronic pain patients into five hierarchical categories: grade 0 for no pain, grade I for low disability-low intensity, grade II for low disability-high intensity, grade III for high disability-moderately limiting, and grade IV for high disability-severely limiting [13].
4. *PainDetect Questionnaire (PDQ)*: Pain DETECT is a screening questionnaire for neuropathic pain. A score above 18 points indicates a neuropathic component of pain, and a score below 13 points suggests that neuropathic pain is unlikely [9].
5. *Central Sensitization Inventory (CSI)*: The Central Sensitization Inventory (CSI) is a self-report outcome measure designed to identify patients with symptoms of CS. The following classification was used: subclinical: 0 to 29, Mild: 30 to 39, Moderate: 40 to 49, Severe: 50 to 59, Extreme: 60 to 100 [14].

### Inclusion and exclusion criteria

Each participant included in the final analyses had to meet the following criteria:

1. Autoimmune arthritis.
2. Age > 16 years old.
3. CPGS grade ≥ I.

Exclusion criteria included:

1. Primary pain syndromes, such as fibromyalgia or complex regional pain syndrome (CRPS), to avoid bias in the CSI.
2. Severe neurological diseases, including multiple sclerosis, limb or facial paralysis, cerebral palsy, amyotrophic lateral sclerosis, epilepsy, polyneuropathy, a history of stroke, and spinal disc disease, to avoid bias in the PDQ.

### Statistical analysis

The distribution of continuous variables was initially assessed using the Shapiro-Wilk test. Normally distributed data were presented as means with standard deviations (SD), while non-normally distributed data were reported as medians with interquartile ranges (IQR). Differences between normally distributed data were analyzed using Welch’s t-test for comparisons between two groups and ANOVA for comparisons among more than two groups. In post-hoc analyses, Tukey’s HSD test was employed for variables found to be significant in ANOVA. Results were reported as mean differences (MD) with 95% confidence intervals (95% CI). Otherwise specified. The Kendall τ coefficient was used in correlation analyses. Significant variables in univariable analysis were included in multivariable linear regression. All statistical analyses were conducted using Python 3.11.5 with the SciPy (version 1.14.1) and statsmodels (version 0.14.4) packages, with p-values less than 0.05 considered statistically significant.

### Ethics

This study employed an anonymous online survey that did not collect any sensitive personal data and involved no risk or intervention for participants. Participants were clearly informed about the study’s design and objectives. By completing the survey, participants provided their consent to participate in the study, as stated in the survey introduction (Appendix 1). The study was conducted in accordance with the principles outlined in the Declaration of Helsinki.

## Results

### Demographic, lifestyle, and clinical characteristics

A total of 231 responses were collected. Fourteen responses were removed due to incomplete questionnaires or non-rheumatological diagnoses. Moreover, sixteen patients reporting fibromyalgia or complex regional pain syndrome, along with another sixteen with severe neurological disorders, were excluded. Ultimately, 185 responses were included in the final analysis—the study group characteristics are presented in Table 1.

**Table 1:** Study group characteristics

Variable	Overall
n	185
Age; mean (SD)	43.2 (11.7)
Gender	
Female	176 (95.1)
Male	9 (4.9)
Diagnosis	
Rheumatoid arthritis	118 (63.7)
Spondyloarthropathies (including psoriatic arthritis)	50 (27)
Unclassified arthritis	14 (7.5)
Mixed connective tissue disease	3 (1.6)
Age at diagnosis; mean (SD)	37.4 (12.1)
Diagnostic delay	
Up to 3 months	37 (20.0)
Up to 6 months	46 (24.8)
Up to 1 year	40 (21.6)
Up to 3 years	30 (16.2)
Up to 5 years	11 (5.9)
Up to 8 years	7 (3.7)
Over 8 years	14 (7.5)
Disease duration (years); median (IQR)	2 (1.0–8.0)
Occupation type	
Sedentary	103 (55.6)
Physically	82 (44.3)
Night shifts	40 (21.6)
Surgical procedure due to a rheumatic disease	34 (18.4)
bDMARDs	43 (23.2)
Osteoarthritis	42 (22.7)
Depression	33 (17.8)
Hypercholesterolemia	29 (15.7)
Number of comorbidities; median (IQR)	2.0 (1.0–3.0)
Additional painkillers	
Without	23 (12.4)
NSAID’s	114 (61.6)
Opioids	48 (25.9)
Co-analgesics (pregabalin, venlafaxine, duloxetine)	18 (9.7)
Frequency of painkillers intake	
Rarer than once a day	111 (60.0)
Once a day	45 (24.3)
More than once a day	29 (15.7)
Smoking	
No	104 (56.2)
Ex-smoker	36 (19.5)
Yes	45 (24.3)
Alcohol consumption	
Not drinking at all	100 (54.1)
< 2 units monthly	41 (22.2)
< 4 units monthly	17 (9.2)
>2 units at once, but < 4 units monthly	3 (1.6)
< 8 units monthly	17 (9.2)
< 16 units monthly	4 (2.2)
> 16 units monthly	3 (1.6)
Physical activity (PA)	
No additional PA	92 (49.7)
PA below the recommended threshold	41 (22.2)
PA within 1–2 times the recommended threshold	21 (11.4)
PA above 2 times the threshold	31 (16.8)
BMI; median (IQR)	23.9 (21.4–27.8)
Chronic Pain Grade Scale	
Grade I	21 (11.4)
Grade II	49 (26.5)
Grade III	49 (26.5)
Grade IV	66 (35.7)
CSI score; median (IQR)	54.0 (45.0–60.0)
CSI classification	
Subclinical	6 (3.2)
Mild	26 (14.1)
Moderate	33 (17.8)
Strong	73 (39.5)
Strong	47 (25.4)
PDQ; mean (SD)	18.5 (6.7)
PDQ classification	
Absent	36 (19.5)
Borderline	43 (23.2)
Present	106 (57.3)

Data are shown as frequencies n (%) unless otherwise specified

*bDMARDs* biologic disease-modifying anti-rheumatic drugs,* CSI* central sensitization inventory,* NSAIDs* non-steroidal anti-inflammatory drugs,* PDQ* PainDetect Questionnaire

### Relationships between CPGS, CSI, and PDQ

**Fig. 1:**
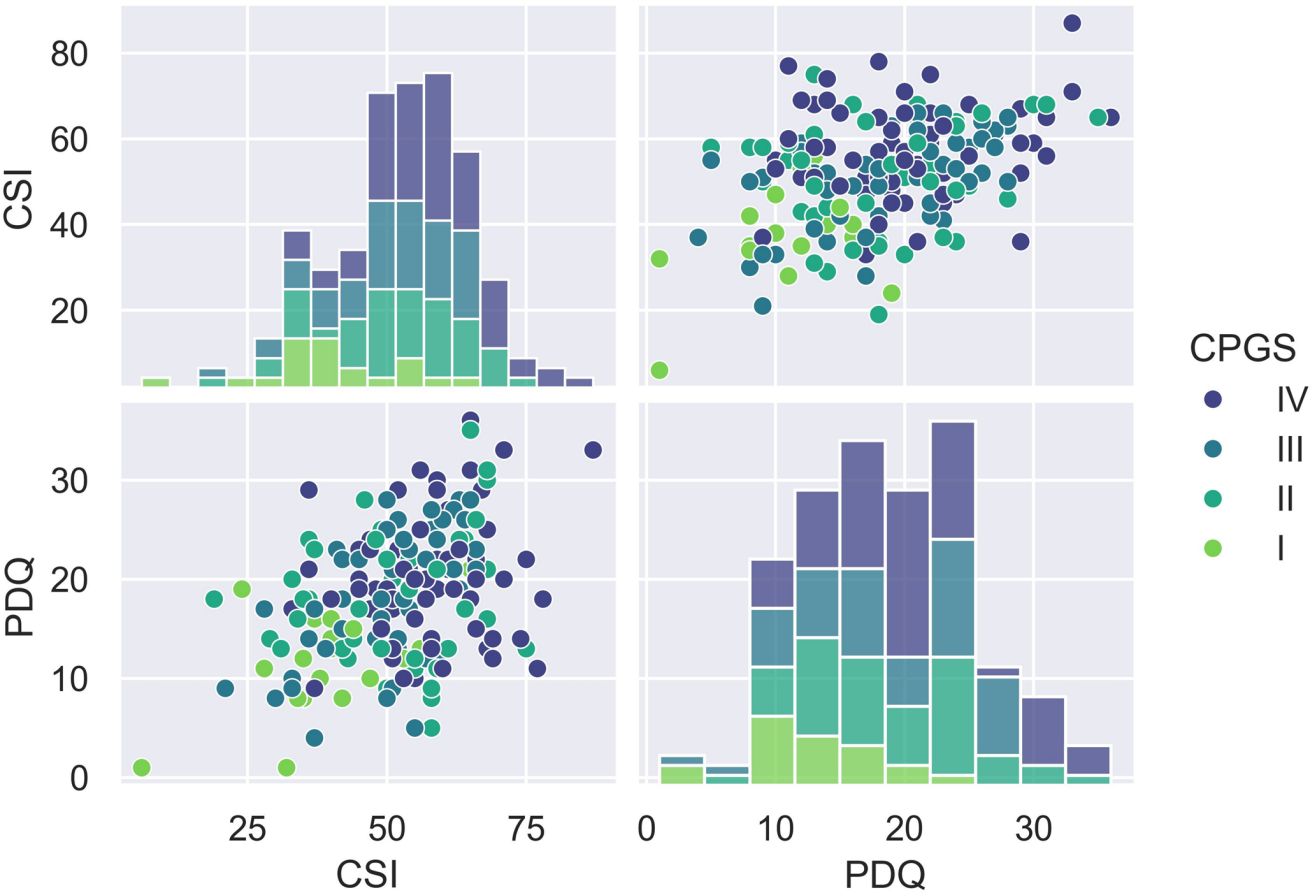
The figure shows the distribution of CSI, PDQ, and CPGS in relation to each other. *CPGS* Chronic Pain Grade Scale, *CSI* central sensitization inventory, *PDQ* PainDetect Questionnaire

Moderate correlations were observed between CSI and PDQ (τ = 0.27, 95%CI = 0.13–0.4, *p* < 0.001), as well as between CPGS and CSI (τ = 0.25, 95%CI: 0.11–0.38, *p* < 0.001) and PD and CPGS (τ = 0.19, 95%CI: 0.05–0.32, *p* < 0.001). We also observed that most participants (86%) who presented with at least a moderate CSI grade (CSI score of at least 40 points) had a positive or borderline grade on PDQ. The results are presented in Fig. 1; Table 2.

**Table 2:** Frequencies n (%) of PDQ and CSI grades concerning each other

		CSI				
		Subclinical	Mild	Moderate	Strong	Very Strong
PDQ	Absent	3 (1.6)	11 (5.9)	3 (1.6)	16 (8.6)	3 (1.6)
	Borderline	1 (0.5)	8 (4.3)	15 (8.1)	11 (5.9)	8 (4.3)
	Present	2 (1.1)	7 (3.8)	15 (8.1)	46 (24.8)	36 (19.4)

Pearson’s χ² (6) = 32.5, Cramér’s V = 0.31, p-value < 0.001. Note: Subclinical CSI was excluded due to sample size

### Comorbidities and lifestyle factors

**Fig. 2:**
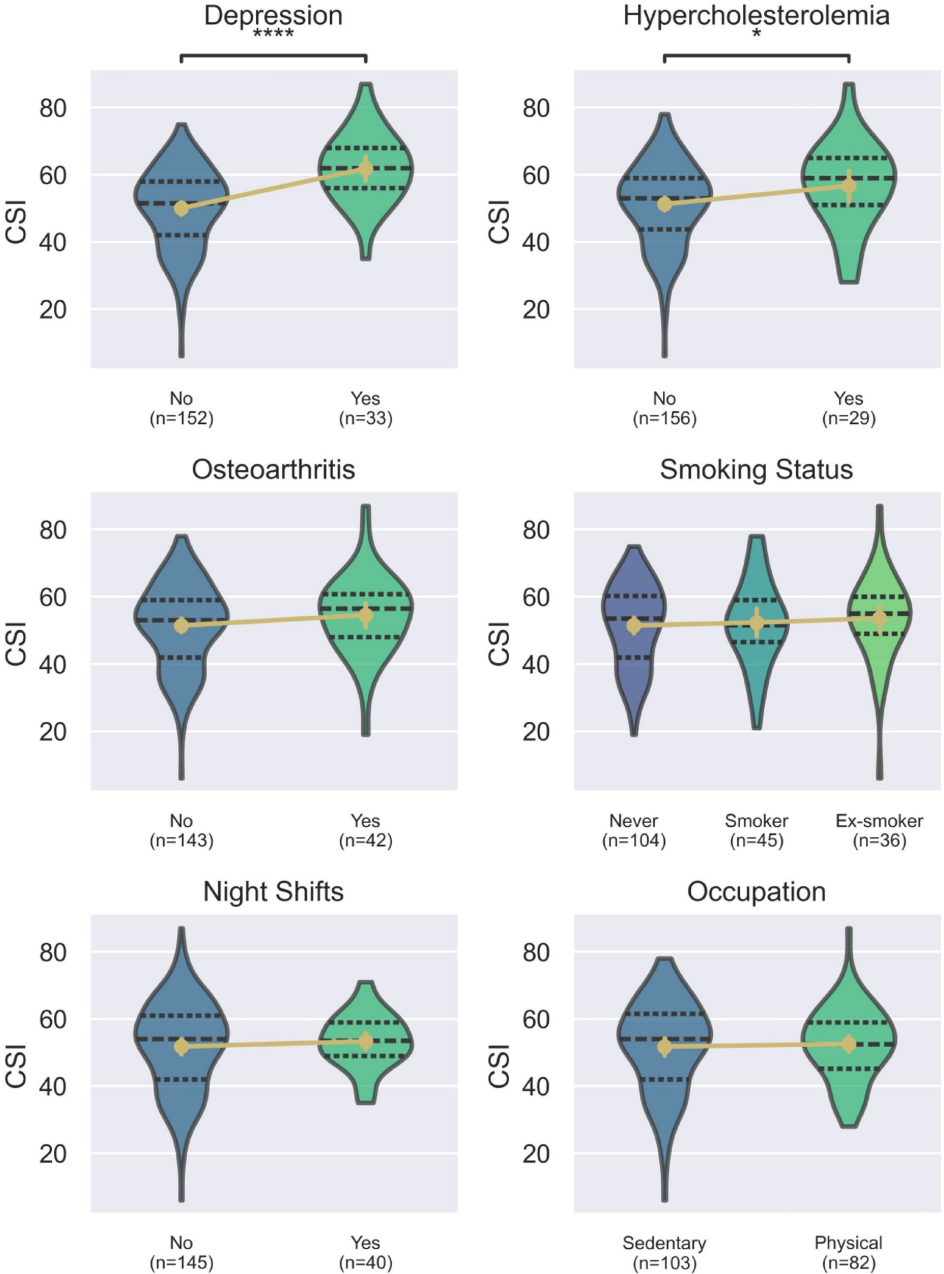
Violin plots represent differences in CSI score according to specified comorbidities or lifestyle factors; Dashed lines represent medians with IQR. The gold points represent means with 95% CI. The p-values are marked as follows: * for *p* < 0.05, ** for *p* < 0.01, *** for *p* < 0.001, and **** for *p* < 0.0001. *CSI* Central Sensitization Inventory, *PDQ* PainDetect Questionnaire

The number of comorbidities correlated with the CSI (τ = 0.16, 95%CI: 0.01–0.29, *p* = 0.005). Among assessed comorbidities, depression (MD = 11.8, 95% CI: 7.7–15.9, *p* < 0.001) and hypercholesterolemia (MD = 5.4, 95% CI: 0.2–10.6, *p* = 0.042) were associated with higher scores on the CSI, where no significant difference was observed in patients with osteoarthritis (MD = 3.1, 95% CI: -1.1–7.3, *p* = 0.14). There was a moderate negative correlation between CSI and mood at participation (τ =-0.23, 95%CI: -0.36 – -0.09, *p* < 0.001) and social interactions (τ =-0.26, 95%CI: -0.39 – -0.12, *p* < 0.001). Across lifestyle factors, a negative correlation was observed between physical activity and CSI (τ = -0.14, 95%CI:-0.28–0.0), *p* = 0.011). Conversely, there was no correlation with alcohol consumption (*p* = 0.60). Notably, 54% of participants reported complete abstinence from alcohol, while 22% indicated consumption of no more than two units per month. Similarly, no statistical differences existed between smoking status (*p* = 0.50) and BMI (*p* = 0.5). There were no differences between sedentary or physically type of occupation (*p* = 0.96), as well as working on night shifts (*p* = 0.36). Detailed results are provided in Fig. 2.

### Clinical features

**Fig. 3:**
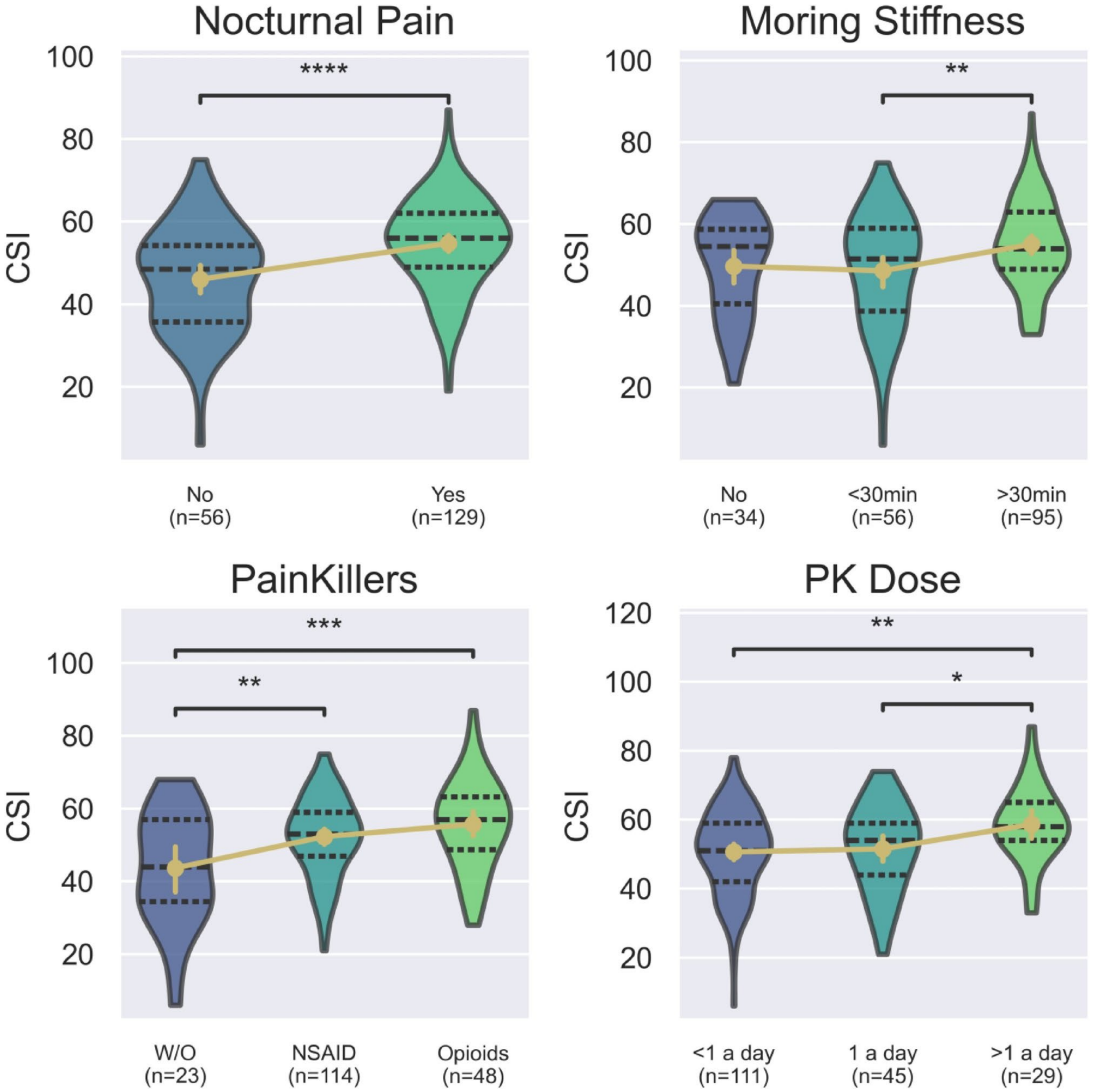
Violin plots represent differences in CSI score according to specific clinical features; dashed lines represent medians with IQR. The gold points represent means with 95% CI. The p-values are marked as follows: * for *p* < 0.05, ** for *p* < 0.01, *** for *p* < 0.001, and **** for *p* < 0.0001. *CSI* Central Sensitization Inventory, *NSAIDs* non-steroidal anti-inflammatory drugs, *PK* painkillers, *PDQ* PainDetect Questionnaire, *W/O* Without

According to clinical diagnosis, there was no significant difference in CSI score between patients with rheumatoid arthritis and spondyloarthropathies (Median (IQR): 53 (42–60) vs. 55 (50–60 ), U_Mann−Whitney_ = 2512, *p* = 0.13). Comparisons that included unclassified arthritis or MCTD were omitted due to the insufficient sample size. Regarding clinical presentation, nocturnal pain was associated with a higher CSI score (MD = 8.7, 95% CI: 4.7–12.7, *p* < 0.001). Similarly, patients with morning stiffness lasting more than 30 min compared to those with less than 30 min (MD = 6.5, 95% CI: 1.7–11.3, p_adj_=0.005). The difference compared to patients without morning stiffness did not reach statistical significance (MD = 5.4, 95% CI: -0.4–11.1, p_adj_ =0.072). The need of additional painkillers, such as opioids (MD = 12, 95% CI: 4.9–19.3, p_adj_ =0.003) or NSAIDs (MD = 8.6, 95% CI: 2.2–15.1, p_adj_ =0.005) were associated with higher CSI score, as well as, frequency of their intake (τ = 0.15, 95%CI: 0.01–0.29, *p* = 0.015). Taking above one dose daily was associated with a higher CSI score compared to once a day (MD = 7.1, 95% CI: 0.3–14, p_adj_ =0.039) and rarer (MD = 8, 95% CI: 2–14, p_adj_ =0.005). Notably, there was no statistical difference between opioids and NSAIDs (MD = 3.4 (95% CI: -1.4–8.3), p_adj_ =0.22). Disease duration was associated with a higher CSI score (τ = 0.12, 95%CI:0.02–0.26, *p* = 0.019). However, there was no association with diagnosis delay (*p* = 0.63) and age at participation (*p* = 0.21). The results are presented in Fig. 3.

### Multivariable analysis

All variables significant in univariable analysis were included in multivariable linear regression. Depression (β = 8.7) and mood (β = -0.9, on a 0–10 scale) remained significant factors contributing to the final CSI score. Nocturnal pain (β = 4.5), need for painkillers (β = 5.3), disease duration above 2 years (β = 3.3), and CPGS grade (β = 2.8, from I to IV grade) remained significant clinical factors. The detailed results are presented in Table 3.

**Table 3:** Multivariable linear regression model

Variable	Coefficient	95% CI	*p*-value
Intercept	42.9	33.4 to 52.4	< 0.001
Depression	8.7	4.5 to 12.8	< 0.001
Nocturnal pain	4.5	0.9 to 8.0	0.013
Need for painkillers	5.3	0.5 to 10.1	0.032
PK dose > 1 a day	0.0	– 3.3 to 3.3	0.99
Disease duration > 2 years*	3.3	0.2–6.4	0.036
Number of comorbidities > 1**	– 1.6	– 4.7 to 1.5	0.31
Hypercholesterolemia	– 0.3	– 4.6 to 4.0	0.88
Moring stiffness > 30 min	2.0	– 1.2 to 5.2	0.23
Social interactions	– 0.6	–1.4 to 0.1	0.1
Mood	– 0.9	–1.8 to – 0.1	0.031
Physical activity	– 0.6	–1.9 to 0.7	0.39
CPGS grade	2.8	1.2 to 4.4	< 0.001

F(12) = 9.8, p-value < 0.001, R^2^ = 40%, R^2^-adjusted = 37%, Skew=-0.11, Kurtosis = 2.94

*The variable was dichotomized based on the median value to enhance the generalizability of the models

**Depression and hypercholesterolemia were excluded from the total number of comorbidities to provide independence of variables, and then were dichotomized based on the median value

## Discussion

In this study, we describe and analyze patients with autoimmune arthritis suffering from chronic pain. Only 3.1% of participants did not exhibit any symptoms of nociplastic pain (CSI < 30 points), and only 17.1% of patients scored below 40 points, which often serves as the threshold for this questionnaire [15]. To the best of our knowledge, this is the first study to assess the impact of clinical and lifestyle factors on nociplastic pain in this patient population.

Our study evokes PDQ and CSI limitations in assessing pain components. Firstly, PDQ seems to be an inappropriate tool for screening neuropathic pain in patients with nociplastic pain. Some studies suggested that patients with fibromyalgia or with centrally mediated pain tend to have higher scores in PDQ [10, 16]. Although those components may coexist, the substantial proportions of patients with at least moderate grade in CSI scored positively in PDQ. Thus, in our opinion, patients suspected of coexistence of these two components need more detailed clinical evaluation. Secondly, we showed that depression and mood during CSI fulfillment, adjusted for other clinical features, have a substantial impact on the result. Assuming neutral mood on 4–6 points, the final score may differ up to approximately 4 points. Together with depression, it gives almost 13 points, which constitute around 60% of the points for clinical features included in our model. Even though depression and mood are part of central sensitization and subsequently nociplastic pain, their effect seems to be overestimated, as noted in other studies [17, 18]. Our study quantified their effects, supporting proper application and interpretation of CSI in practice.

Furthermore, we showed that nocturnal pain and pain-related disability (CPGS) have an essential impact on the development of nociplastic pain. The former may be associated with high disease activity and inflammation contributing to nociceptive pain and further sensitization, as well as be linked to sleep deprivation, a factor that has been shown to accelerate central sensitization [19]. However, it is impossible to determine from our study whether this effect is solely related to sleep deprivation or extends beyond it. The ISAP algorithm mentions only sleep deprivation [8], in our opinion, questions about nocturnal pain may be even easier to implement in the screening of nociplastic pain. Similarly, assessment of pain-related disability may be more objective as compared to reported chronic pain intensity, which is usually overestimated in those patients. Our analysis showed that patients with high disability are more likely to develop nociplastic pain, therefore screening in this cohort may be particularly beneficial.

Lastly, the results presented in our study suggest that diagnosis and management of nociplastic pain are insufficient. Patients with higher nociplastic pain were taking painkillers more frequently, and the number of patients taking opioids was substantial (26%). Only 10% of patients received pregabalin, venlafaxine, or duloxetine, which should be considered in the nociplastic component. Even though our understanding and management of nociplastic pain is limited, we should educate both physicians and patients about this phenomenon to minimize the social and health costs of rheumatic diseases.

### Limitations

Despite the utmost care in data collection and analysis, our study has some limitations that need to be highlighted. Firstly, online data collection enabled detailed characteristics of a relatively large cohort of patients with nociplastic pain, however, it precluded controlling the completion process. Consequently, assessing each participant’s understanding of the questions was not feasible. Nevertheless, each question included a blank space for open responses, allowing participants to describe their conditions if none of the closed responses fit. This enabled us to eliminate the most suspicious responses and account for some comorbidities to minimize bias. Moreover, our results are consistent with similar studies conducted in clinical settings, suggesting their potential real-life application. Secondly, there are some objections regarding measuring nociplastic pain using CSI. Following ISAP criteria, (1) chronic pain was assessed by CPGS; each patient above grade I met this criterion. The recent meta-analysis demonstrated consistency between CSI and quantitative sensory testing (QST) [20] This led to the conclusion that a patient with a high CSI score meets criteria (2) and (3). Potential biases were included in the analysis to provide more reliable results. Despite its limitations and lack of gold standard in diagnosis, the CSI appears to be a valid screening tool for neoplastic pain, particularly within our study design.

### Further research

There is still room for improvement in the diagnosis and management of nociplastic pain. If chronic pain and inflammation contribute to sensitization, which may vary depending on individual predispositions, educating patients—even at the time of diagnosis—could be beneficial for long-term management as a prophylactic method. However, the form of education and its impact need to be investigated.

## Conclusions

The PainDetect questionnaire (PDQ) may not be an appropriate tool for screening neuropathic pain in patients with high levels of nociplastic pain. A low mood during the completion of the Central Sensitization Inventory (CSI) and concurrent depression may significantly bias the final score and should be considered when interpreting the results. The assessment of nocturnal pain and pain-related disability may serve as practical and easily applicable risk factors complementary to the ISAP criteria for screening patients with nociplastic pain.

## Supplementary Information

Below is the link to the electronic supplementary material.

Supplementary Material 1

Supplementary Material 2

## Data Availability

The data underlying this article will be shared on request to the corresponding author.
