# Supplementary material for: Risk factors of nociplastic pain in patients with autoimmune arthritis: web-based cross-sectional survey of patients
Source: Rheumatol Int. 2025 Aug 22;45(9):205. doi: 10.1007/s00296-025-05948-7 (PMC12373665; doi:10.1007/s00296-025-05948-7)
Supplement: Supplementary file 1 — Supplementary Material 1 [file 296_2025_5948_MOESM1_ESM.pdf]

Introduction

Dear Sir/Madam,

The following survey was created by doctors and students from the Department of Rheumatology at the Medical University of Łódź. The aim of the study is to determine whether various risk factors and the course of treatment influence the occurrence of nociplastic pain in patients with arthritis.

The study will be published in an international journal, and your participation may help improve future care and treatment for patients with rheumatic diseases.

Your answers are anonymous, and no data other than what you provide in the survey will be collected or stored.

Note: Each reported disease has to be diagnosed by a doctor

By completing and submitting the survey, you consent to participate in the study.

Survey Questions

1. How would you rate your mood today? (0 – 10)

- Scale: Very bad/negative (0) to Very good/positive (10)

2. How old are you?

3. Sex

- Male
- Female

4. Place of residence

- Village
- City up to 20,000 inhabitants
- City up to 50,000 inhabitants
- City up to 100,000 inhabitants
- City up to 500,000 inhabitants
- City over 500,000 inhabitants
- Warsaw

5. What is your education level?

- Primary

- Vocational
- Secondary
- Bachelor/Engineer
- Master's degree or higher

6. What medical condition(s) have you been diagnosed with by a doctor?

- Rheumatoid Arthritis (RA)
- Psoriatic Arthritis (PsA)
- Ankylosing Spondylitis (AS)
- Undifferentiated Spondyloarthropathy
- Undifferentiated Arthritis
- Other

7. At what age were you diagnosed with the disease?

8. How long was the time between symptom onset and diagnosis/start of treatment?

- Up to 3 months
- Up to 6 months
- Up to 12 months (1 year)
- Up to 3 years
- Up to 5 years
- Up to 8 years
- More than 8 years

12. Do you also suffer from any of the following? (diagnosed by doctor)

- Osteoarthritis
- Fibromyalgia
- Complex Regional Pain Syndrome (CRPS)
- No

13. Have you been diagnosed with osteoporosis?

- Yes
- No

14. Do you take any of the following medications? (multiple options possible)

- Vitamin D3
- Bisphosphonates
- Denosumab
- Romosozumab

- Teriparatide
- No

15. Have you had surgery due to a rheumatic disease?

- Yes
- No

16. Do you regularly take medication for arthritis?

- Yes
- No, I forget several times a week
- No, I forget several times a month
- No, I forget several times a year
- I'm not on longterm treatment

17. Do you experience joint stiffness and/or pain in the morning upon getting out of bed?

- Yes, lasts more than 30 minutes
- Yes, lasts up to 30 minutes
- No

18. Do you experience joint pain at night?

- Yes
- No

19. How does physical activity affect your joint pain?

- Gradually relieves pain
- Gradually worsens pain
- No effect

20. At what time of day is your joint pain the worst?

- Morning
- Midday
- Afternoon
- Evening
- Night

21. Has any of the listed medications been prescribed to you by a rheumatologist for joint pain?

- Pregabalin (Lyrica, Alzipram, Lyrica Genoptim, Pregabalin Zentiva)
- Gabapentin (Neurontin, Gabapentin Teva, Gabapentin Sandoz)
- Duloxetine (Cymbalta, Dulsevia, Dulofor)

- None

22. Do you take any additional medications to relieve joint pain?
- Nonsteroidal antiinflammatory drugs (e.g., Ibuprofen, Ketoprofen, Diclofenac)
- Paracetamol (e.g., Apap)
- Tramadol (e.g., Tramal)
- None
- Other

23. How often do you take the abovementioned medications to relieve joint pain?
- Several times a day
- Once a day
- Several times a week
- Several times a month
- Several times a year
- I do not take

24. Please enter your height in cm (optional if unknown)

25. Please enter your weight in kg (optional if unknown)

26. What type of work do you do?
    - Sedentary (mental work)
    - Active: physical work
    - Active: sedentary work
    - Not working: I do not work
    - Not working: previously physical work
    - Not working: previously mental work

27. Are you able to perform your job?
    - Yes, without problems
    - Yes, with moderate limitations
    - Yes, but I had to change jobs due to the disease
    - No
    - Not applicable (e.g., retired)

28. How do you assess the frequency and quality of your interactions with others (family, friends)?
    (VAS Scale from 0 to 10)
    - 0: Very much alone/isolated
    - 10: Very frequent with support (help from loved ones)

29. Do you use private medical care services?
    - Yes

- No

30. Do you have any other autoimmune diseases?
    - Type 1 diabetes
    - Hashimoto’s disease
    - GravesBasedow disease
    - Sjögren’s syndrome
    - Crohn’s disease
    - Ulcerative colitis
    - Psoriasis
    - None
    - Other
31. Do you have any of the following conditions?
    - Type 2 diabetes
    - Hypertension
    - Hypercholesterolemia (high cholesterol)
    - None

32. Do you have any lung diseases?
    - Chronic obstructive pulmonary disease (COPD)
    - Interstitial lung disease
    - Asthma
    - None
    - Other
33. Do you have any cardiovascular diseases?
    - Chronic hypertension
    - Heart failure
    - Coronary artery disease
    - Peripheral artery disease (atherosclerosis)
    - None
    - Other
34. Do you suffer from any mental health conditions?
    - Depression
    - Posttraumatic stress disorder (PTSD)
    - Bipolar disorder
    - Anxiety
    - None
    - Other
35. Do you have any neurological conditions?
    - History of stroke
    - Dementia (e.g., Alzheimer’s disease, frontotemporal dementia)
    - Parkinson’s disease
    - Epilepsy

- None
- Other

36. Do you take any of the following antidepressant medications?
    - Sertraline (Zoloft, Asentra, Sertraline Klorid)
    - Citalopram (Celexa, Citalopram Actavis)
    - Escitalopram (Lexapro, Escit, Eloxat, Eloxetin, Depralin Actavis)
    - Paroxetine (Paxil, Parogen, Xetane)
    - Venlafaxine (Effexor XR, Velaxin, Venlafaxine Buflox, Effexin ER)
    - Mirtazapine (Remeron, Mitor, Mirtagen, Mirtaxin)
    - Mianserin (Luvox, Depaxole)
    - None
    - Other
37. Do you smoke cigarettes?
    - Yes
    - Never smoked
    - I don’t smoke (quit for at least a month)
    - A household member smokes

38. Do you regularly consume alcohol?
    - Yes, more than 2 beers (500ml) or more than 2 glasses of wine (200ml) or more than 3 shots of vodka (180ml) daily or almost daily
    - Yes, 2 beers or 2 glasses of wine or up to 3 shots of vodka daily or almost daily
    - Yes, up to 2 beers or 2 glasses of wine or up to 3 shots of vodka once a week
    - Yes, more than 2 beers or 2 glasses of wine or up to 3 shots of vodka 1–2 times a week (e.g., weekends)
    - Yes, a few times a year, maximum 4 times a month
    - Yes, irregularly a few times a year, maximum 1–2 times a month
    - Yes, occasionally, but in amounts exceeding 2 beers or 2 glasses of wine or 3 shots of vodka
    - I do not drink alcohol
39. Do you follow any of the following diets/nutritional lifestyles?
    - Vegetarian diet
    - Vegan diet
    - Glutenfree diet
    - Lactosefree diet
    - None
    - Other
40. Do you practice intermittent fasting (e.g., 16:8, 16 hours fasting, 8 hours eating window)?
    - Yes
    - No
41. Physical activity level
    - Over 5 hours of moderate activity or over 2 hours of intense activity weekly

- Over 2.5 hours of moderate activity or over 1.5 hours of intense activity weekly
- Below 2 hours of moderate activity or below 1 hour 15 minutes of intense activity weekly
- No additional activities weekly

42. Do you work night shifts?

- Yes
- No

Chronic Pain Grade Scale (CPGS) Questionnaire

Pain Assessment

1. **Current Pain Intensity**:

   How would you rate your pain on a 0-10 scale at the present time (right now)?

   Scale: 0 = 'No pain', 10 = 'Pain as bad as it could be'

   ○ 0. ○ 1. ○ 2. ○ 3. ○ 4. ○ 5. ○ 6. ○ 7. ○ 8. ○ 9. ○ 10.

2. **Worst Pain in the Past 6 Months**:

   In the past 6 months, how intense was your worst pain rated on a 0-10 scale?

   ○ 0. ○ 1. ○ 2. ○ 3. ○ 4. ○ 5. ○ 6. ○ 7. ○ 8. ○ 9. ○ 10.

3. **Average Pain Intensity**:

   In the past 6 months, on average, how intense was your pain rated on a 0-10 scale?

   Consider your usual pain at times you were experiencing pain.

   ○ 0. ○ 1. ○ 2. ○ 3. ○ 4. ○ 5. ○ 6. ○ 7. ○ 8. ○ 9. ○ 10.

Impact of Pain on Activities

1. **Impact on Usual Activities**:

   - About how many days in the last 6 months have you been kept from your usual activities (work, school, housework) because of this pain?
   - ______________________

2. **Interference with Daily Activities**:

In the past 6 months, how much has this pain interfered with your daily activities?

Scale: 0 = 'No interference', 10 = 'Extreme change'

○	○	○	○	○	○	○	○	○	○	○
0.	1.	2.	3.	4.	5.	6.	7.	8.	9.	10.

3. **Change in Recreational, Social, and Family Activities**:

In the past 6 months, how much has this pain changed your ability to take part in recreational, social, and family activities?

Scale: 0 = 'No change', 10 = 'Extreme change'

○	○	○	○	○	○	○	○	○	○	○
0.	1.	2.	3.	4.	5.	6.	7.	8.	9.	10.

4. **Change in Ability to Work (Including Housework)**:

In the past 6 months, how has this pain changed your ability to work (including housework)?

Scale: 0 = 'No change', 10 = 'Extreme change'

○	○	○	○	○	○	○	○	○	○	○
0.	1.	2.	3.	4.	5.	6.	7.	8.	9.	10.

[illegible] ____________

Reference: Von Korff M, Ormel J, Keefe FJ, Dworkin SF. Grading the severity of chronic pain. Pain, 1992; 50: 133-49.

painDETECT PAIN QUESTIONNAIRE

Date: ____________	Patient:	Last name: ______________	First name: ______________

How would you assess your pain **now**, at this moment?

0	1	2	3	4	5	6	7	8	9	10

none max.

How strong was the **strongest** pain during the past 4 weeks?

0	1	2	3	4	5	6	7	8	9	10

none max.

How strong was the pain during the past 4 weeks **on average**?

0	1	2	3	4	5	6	7	8	9	10

none max.

Mark the picture that best describes the course of your pain:

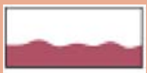
Persistent pain with slight fluctuations ☐

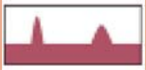
Persistent pain with pain attacks ☐

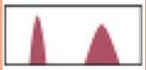
Pain attacks without pain between them ☐

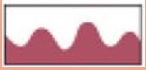
Pain attacks with pain between them ☐

Please mark your
main area of pain

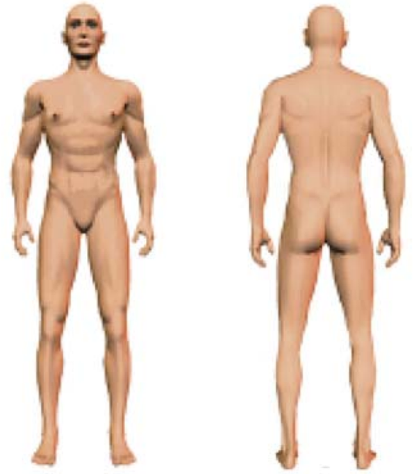

Does your pain radiate to other regions of your body? yes ☐ no ☐

If yes, please draw the direction in which the pain radiates.

Do you suffer from a burning sensation (e.g., stinging nettles) in the marked areas?

never ☐ hardly noticed ☐ slightly ☐ moderately ☐ strongly ☐ very strongly ☐

Do you have a tingling or prickling sensation in the area of your pain (like crawling ants or electrical tingling)?

never ☐ hardly noticed ☐ slightly ☐ moderately ☐ strongly ☐ very strongly ☐

Is light touching (clothing, a blanket) in this area painful?

never ☐ hardly noticed ☐ slightly ☐ moderately ☐ strongly ☐ very strongly ☐

Do you have sudden pain attacks in the area of your pain, like electric shocks?

never ☐ hardly noticed ☐ slightly ☐ moderately ☐ strongly ☐ very strongly ☐

Is cold or heat (bath water) in this area occasionally painful?

never ☐ hardly noticed ☐ slightly ☐ moderately ☐ strongly ☐ very strongly ☐

Do you suffer from a sensation of numbness in the areas that you marked?

never ☐ hardly noticed ☐ slightly ☐ moderately ☐ strongly ☐ very strongly ☐

Does slight pressure in this area, e.g., with a finger, trigger pain?

never ☐ hardly noticed ☐ slightly ☐ moderately ☐ strongly ☐ very strongly ☐

CSI Inventory (Part A)

Please circle the best response to the right of each statement.
Key for Scoring: **Never** = **0**, **Rarely** = **1**, **Sometimes** = **2**, **Often** = **3**, **Always** = **4**

1. I feel tired and unrefreshed when I wake from sleeping.	Never	Rarely	Sometimes	Often	Always
2. My muscles feel stiff and achy.	Never	Rarely	Sometimes	Often	Always
3. I have anxiety attacks.	Never	Rarely	Sometimes	Often	Always
4. I grind or clench my teeth.	Never	Rarely	Sometimes	Often	Always
5. I have problems with diarrhea and/or constipation.	Never	Rarely	Sometimes	Often	Always
6. I need help in performing my daily activities.	Never	Rarely	Sometimes	Often	Always
7. I am sensitive to bright lights.	Never	Rarely	Sometimes	Often	Always
8. I get tired very easily when I am physically active.	Never	Rarely	Sometimes	Often	Always
9. I feel pain all over my body.	Never	Rarely	Sometimes	Often	Always
10. I have headaches.	Never	Rarely	Sometimes	Often	Always
11. I feel discomfort in my bladder and/or burning when I urinate.	Never	Rarely	Sometimes	Often	Always
12. I do not sleep well.	Never	Rarely	Sometimes	Often	Always
13. I have difficulty concentrating.	Never	Rarely	Sometimes	Often	Always
14. I have skin problems such as dryness, itchiness, or rashes.	Never	Rarely	Sometimes	Often	Always
15. Stress makes my physical symptoms get worse.	Never	Rarely	Sometimes	Often	Always
16. I feel sad or depressed.	Never	Rarely	Sometimes	Often	Always
17. I have low energy.	Never	Rarely	Sometimes	Often	Always
18. I have muscle tension in my neck and shoulders.	Never	Rarely	Sometimes	Often	Always
19. I have pain in my jaw.	Never	Rarely	Sometimes	Often	Always
20. Certain smells, such as perfumes, make me feel dizzy and nauseated.	Never	Rarely	Sometimes	Often	Always
21. I have to urinate frequently.	Never	Rarely	Sometimes	Often	Always
22. My legs feel uncomfortable and restless when I am trying to go to sleep at night.	Never	Rarely	Sometimes	Often	Always
23. I have difficulty remembering things.	Never	Rarely	Sometimes	Often	Always
24. I suffered trauma as a child.	Never	Rarely	Sometimes	Often	Always
25. I have pain in my pelvic area.	Never	Rarely	Sometimes	Often	Always
Total Each Column					

Overall Total

Copyright © 2017, Martha Teater & Don Teater. *Treating Chronic Pain*. All rights reserved.
