# Supplementary figures and images for: Risk factors of nociplastic pain in patients with autoimmune arthritis: web-based cross-sectional survey of patients
Source: Rheumatol Int. 2025 Aug 22;45(9):205. doi: 10.1007/s00296-025-05948-7 (PMC12373665; doi:10.1007/s00296-025-05948-7)

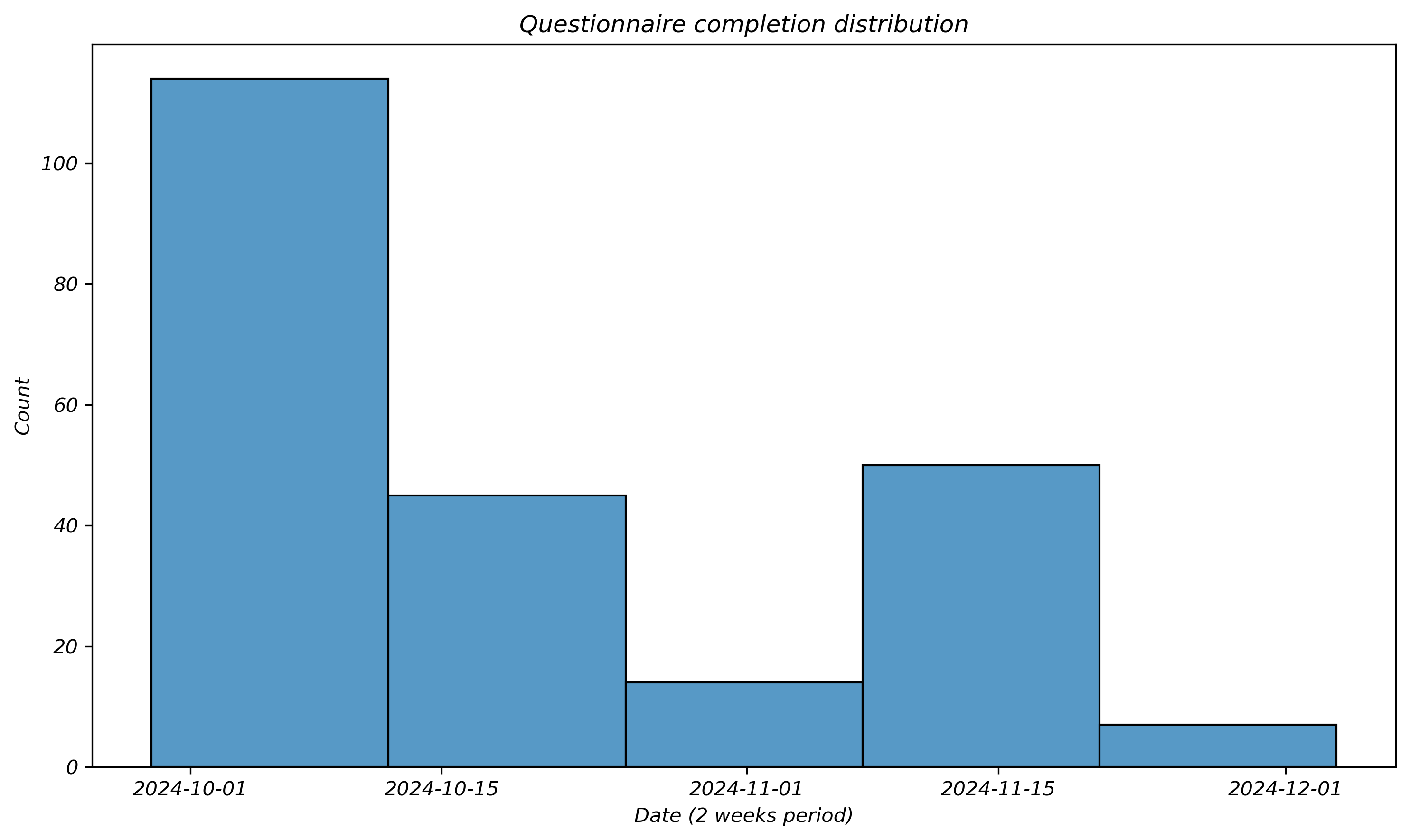

Supplement: Supplementary file 2 — Supplementary Material 2 [file 296_2025_5948_MOESM2_ESM.png]
